# Knowledge, attitudes and preventive practices for human Papilloma virus infection among female sex workers in Lagos metropolis

**DOI:** 10.11604/pamj.2020.36.278.17912

**Published:** 2020-08-14

**Authors:** Nforbih Emile Shu, Abdul-Hakeem Olatunji Abiola, Babatunde Abdulmajeed Akodu, Benjamin Afahakan Bassey, Nadine Misago

**Affiliations:** 1 Department of Community Health and Primary Care, University of Lagos, Lagos, Nigeria,; 2 Faculty of Medicine and Biomedical Sciences, University of Yaoundé I, Yaoundé, Cameroon,; 3 Batié Medical Health Centre, Batié, Western Region, Cameroon,; 4 Lagos University Teaching Hospital, Lagos, Nigeria,; 5 University of Uyo Teaching Hospital, Uyo, Nigeria,; 6 Faculty of Medicine, University of Burundi, Bujumbura, Burundi

**Keywords:** Female sex workers, human Papilloma virus, Lagos

## Abstract

**Introduction:**

risky and hard-to-reach populations like female sex workers (FSW) face a huge burden with sexually transmitted infections (STIs) among which is human Papilloma virus (HPV) infection. This study was conducted to evaluate the knowledge, attitudes and preventive practices for HPV infection among FSW in Lagos, Nigeria.

**Methods:**

a descriptive cross-sectional study was carried out among 403 respondents. The sampling units were FSW in brothels in two urban communities of Lagos. A multistage sampling technique was used for selection of respondents. Pre-tested, validated questionnaire was used for data collection. Responses to knowledge, attitude and practice questions were scored graded as poor (<50%) and good (≥50%). Bivariate analysis were carried out using Chi-square, Fisher exact test and student t-test. Logistic regression model was used for multivariate analysis. P-value < 0.05 was considered statistically significant.

**Results:**

the mean age of the respondents was 32.97 ± 8.43. Majority of the respondents were within the age range of 18-34 years (51.61%), christians (51.12%), single (42.93%) and had secondary education (52.61%). Among the respondents 51.61% had good knowledge, 97.27% had good attitude and 62.28% had good preventive practice. FSW belonging to the age group 35-51 or 52-68 years, were more likely to have a good knowledge compared to those between 18-34 years. FSW with no formal education or living with a relative are less likely to have a good knowledge, compared to those having primary education or living alone. FSW with traditional or other religious beliefs are less likely to have good preventive practices against HPV compared to christian religious belief. Having tertiary education or married makes a FSW less likely, while being widowed makes her more likely to have good preventive practice. FSW living with friends are more likely to be exposed to good preventive practices compared to those living alone.

**Conclusion:**

there is a need for regular health education program on HPV for FSW in order to increase their awareness and encourage best preventive practices against HPV.

## Introduction

Human Papillomavirus (HPV) continues to be a major global public health problem. The double stranded DNA virus is sexually transmissible and also the principal causative agent of cervical cancer [1]. Globally, cervical cancer is the fourth most prevalent cancer in women with approximately 570 000 cases and 311 000 deaths reported in 2018 with low-middle income countries bearing the greatest burden [2]. Several genotypes of HPV have the potential to cause either benign or virulent lesions but HPV type 16 and 18 have been implicated in about 70% of all invasive cervical cancer worldwide [3]. In Nigeria, about 53.1 million women aged greater or equal to 15 years were at risk of cervical cancer with an annual number of 14943 cases of cervical cancer and 10403 cases of deaths in 2019 with the prevalence of HPV 16 and or HPV 18 amongst women with cervical cancer being 66.9% [4]. Despite approval by the World Health Organization (WHO) to use prophylactic vaccines to prevent and control HPV infections (especially genotypes 16 and 18) [5], the burden of HPV infection remains huge in sub-Saharan African countries where the prevalence is high with many asymptomatic carriers of the vaginal, anal and oro-pharyngeal lesions [6, 7].

Key populations such as female sex workers (FSW) and men who have sex with men (MSM) are high risk groups who are vulnerable to many sexually transmissible infections including HPV and HIV [8]. Evidence from studies showed that the prevalence of HPV infection is higher among FSW compared to women in the general population [9-11]. Likewise, knowledge on HPV, HPV vaccines as well as uptake of Pap smear among FSW is poor [12]. High-risk sexual practice and being of younger age are identified as HPV risk factors [13]. The risk of acquiring new HPV infection is increased among FSW who are living with HIV [14,15]. Despite the existing gaps in literature found in other countries in knowledge on HPV, little is known on knowledge, attitudes and preventive practices on HPV among FSW in Nigeria. This study was therefore conducted to evaluate the level of knowledge, attitudes and preventive practices against HPV among FSW in Ikeja and Mushin local government areas of Lagos state, Nigeria. A good appraisal of this will give us a good idea towards evidence-based public health intervention strategies.

## Methods

**Study area:** Lagos state is located in the south western part of Nigeria. It is bounded in the south by a 180 km Atlantic coastline, in the north-east by Ogun state and in the west by the republic of Benin. Lagos state is a rapidly developing city and it´s the commercial and economic capital of Nigeria with a total of 20 recognized local government areas with 16 urban Local Government Area (LGA) and 4 rural LGA. In 2006 following a demographic health survey it had a population of 9,113,605 people [16] and with an annual growth rate of 3.2 the national population commission estimated it at 21 million in 2016. Following results obtained from an HIV epidemic appraisal conducted among FSW in 2012, there were 46,691 FSW with Ikeja and Mushin LGA having 2,871 and 2,940 FSW respectively [17].

**Study design/participants:** a descriptive cross-sectional study design was used. With prevalence (p) of 36.5% of those who had good knowledge in HPV in previous study [18], a sample size of 403 was calculated using statistical formula for descriptive study (n = Z^2^pq/d^2^when target population is ≥ 10,000 and nf = n/1+ (n/N) for populations < 10000). It was then evenly distributed among the two selected LGAs. With the help of the health educators from each LGA secretariat a list of all brothels in each LGA was obtained following community mapping that had been done during former HIV/AIDs intervention programs in each LGA. Local community guides were used to locate all the functional brothels with FSW in each LGA. Several advocacy visits to the brothel managers and FSW chair ladies were then carried out. The inclusion criteria was FSW who were greater or equal to 18 years of age while the exclusion criteria was FSW who have spent less than a month in the occupation.

**Sampling method:** the units of selection were the FSW in each room. Multi-staged sampling method was used for selection of respondents as follows: in stage one, two of the 16 urban LGAs in Lagos state (Ikeja and Mushin LGAs) were selected by simple random sampling method using balloting procedures. In stage two, six brothels were selected from each selected LGA by simple random sampling method using balloting procedure. In stage three, 403 rooms were selected from the list of rooms in the selected brothel by simple random sampling method using balloting procedure. The occupant of each selected room was then enrolled into the study. If the occupant was below 18 years or refused to participate, the next room was selected. Selected rooms with unavailable respondents were revisited by the researchers following booked appointments.

**Data collection:** a pre-tested, structured, interviewer-administered questionnaire with open and closed ended questions was used for collecting the respondent´s responses. The questionnaire was compiled and modified from questionnaires used in studies conducted among FSW in Turkey and Vietnam [19, 20]. With some additional questions added. The questionnaire consisted of four sections: socio-demographic and occupational characteristics, eleven knowledge-based questions, fourteen attitude-based question and eight preventive practices-based question. The questionnaire was pre-tested among 30 randomly selected FSW in a brothel located in another local government area from our study sites (Surulere LGA) to ensure comprehension, validity and reliability. The questions on knowledge, attitude and preventive practices were scored. Each correct response to the knowledge and preventive practices questions were scored one point and any wrong or non-response zero point. Questions on attitudes was scored based on a 5-point Likert scale. Based on literature a cut off score of 50% was used [21]. The total score obtained by each respondent was converted to percentage and scores < 50% were graded as poor and scores ≥ 50% as good.

**Analysis:** data analysis were carried out using Microsoft Excel, Epi info version 7.1.1.14, WinPepi and GraphPad Instat computer statistical software packages. For bivariate analysis, Chi-square and Fisher exact test were used to compare differences between proportions while student t-test was used for comparison of differences between means. For multivariate analysis, logistic regression was performed to evaluate the strength of associations and the overall effects of the independent variables on the dependent variables. P-value ≤ 0.05 was considered statistically significant.

**Ethical consideration:** ethical approval for the research was obtained from the Health Research and Ethics Committee (HREC) of the Lagos University Teaching Hospital. Verbal/written informed consent was obtained from each respondent after having been clearly intimated with the objectives, methodology, advantages and risk involved in the study. Participation was voluntary throughout the study and any participant was free to decline participating in the study at any time without any prejudice. Participants were treated equally irrespective of their social status and other related status. Confidentiality and privacy were ensured by using no identification information.

## Results

Most of the FSW were within the age-group of 18-34 years (51.61%) of age with mean 32.97 ± 8.43 years, christians (51.12%), had secondary education (52.61%), single (42.93%), were Yoruba (39.21%), formerly lived in an urban setting (64.52) (Table 1), had a sex work duration of 13-60 months (53.85%) with median duration 48 months (IQR: 27-72), had a monthly income within 11,000-50,000 naira (60.79%), were active smokers (60.79%) and had within 6-10 (33.75%) sexual partners per week.

**Table 1:** socio-demographic characteristics of female sex workers

Variable (n=403)	Frequency (%)
**Age Group**	
18-34	208 (51.61)
35-51	158 (39.21)
52-68	10 (2.48)
Not given	27 (6.70)
**Mean ± SD = 32.97 ± 8.43**	
Religion	
Christianity	206 (51.12)
Islam	107 (26.55)
Traditional	68 (16.87)
Others	22 (5.46)
**Educational Status**	
None	28 (6.95)
Primary school	121 (30.02)
Secondary school	212 (52.61)
Tertiary Institution	42 (10.42)
**Current Marital Status**	
Single	173 (42.93)
Married	43 (10.67)
Separated	81 (20.10)
Divorced	75 (18.61)
Widowed	31 (7.69)
**Former Habitat**	
Rural	143 (35.48)
Urban	260 (64.52)
**Person Living With**	
Alone	170 (42.18)
Husband	29 (7.20)
Boyfriend	39 (9.68)
Friends	117 (29.03)
Relatives	48 (11.91)
**Tribe**	
Yoruba	158 (39.21)
Igbo	140 (34.74)
Hausa	57 (14.14)
Others	48 (11.91)

**Knowledge of HPV infection among FSW:** half of the FSW had a good knowledge (51.61%) with a percentage mean knowledge score of 45.68±24.94 (Table 2). Most of the FSW knew HPV was sexually transmitted (76.18%), it causes cervical cancer (66%), it could be prevented (50.87%), proper use of condoms could prevent the infection (54.84%) and that receiving the HPV vaccine could prevent the infection (48.64%). However, only a few knew HPV infection doesn´t present with vaginal itches and discharge (19.35%), that Pap smear was used for screening and detection of HPV (38.21%) and that women who had received HPV vaccine should still do a Pap smear (14.64%). A statistically significant association was found between the respondents knowledge grade on HPV infection and their level of education (p = 0.025), their age (p < 0.001), marital status (p = 0.035), tribe (p = 0.035) and their monthly income (p = 0.0003). Most interestingly, the proportion of FSW who had good knowledge about HPV was significantly higher among condom users, compared to non-users (p = 0.015) and among those who undertook a Pap smear screening test, compared to those who have not taken the test (p < 0.001) (Table 3).

**Table 2:** scoring and grading of FSW knowledge, attitudes and preventive practices

Scores and Grade	Knowledge	Attitude	Preventive practice
Scores	Mean ± SD	Mean ± SD	Mean ± SD
Mean score (%)	45.68 ±24.94	721.6699 ±11.664	56.48 ±27.69
**Grade**	**Frequency (%)**	**Frequency (%)**	**Frequency (%)**
Poor	195 (48.39)	1144 (2.7310.92)	152 (37.72)
Good	208 (51.61)	39259 (97.2789.08)	251 (62.28)
Total	403 (100)	403 (100)	403 (100)

**Table 3:** effects of knowledge and attitude grade on condom use and Pap smear screen

Grade	Preventive practices practices		Statistics/p-value
	Yes	No	
	Frequency (%)	Frequency (%)	
**Knowledge grade**	**Condom use**		
Poor	133 (68.21)	62 (31.79)	X^2^=5.89;df=1;p=0.015
Good	165 (79.33)	43 (20.67)	
Total	298 (147.57)	105 (52.46)	
	**Pap smear screening**		
Poor	43 (22.05)	152 (77.95)	X^2^=35.96;df=1;p<0.001
Good	107 (51.44)	101 (48.56)	
Total	150 (73.49)	253 (126.51)	
**Attitude grade**	**Condom use**		
Poor	6 (54.55)	5 (45.45)	X^2^=2.21;df=1;p=0.137
Good	292 (74.49)	100 (25.51)	
Total	298 (129.04)	105 (70.96)	
	**Pap smear screening**		
Poor	0 (0.00)	11 (100)	X^2^=6.705;df=1;p=0.0096
Good	150 (38.27)	242 (61.73)	
Total	150 (38.27)	253 (161.73)	

**Attitudes towards HPV infection among FSW:** most of the FSW had a good attitude (97.27%) with a percentage mean attitude score of 72.66 ± 11.61 (Table 2). The study found, 29.03% and 28.54% had a strong concern of being affected by cervical cancer and genital warts respectively. About 27.05% respondents strongly agreed having cervical cancer will affect their daily activities. 28.54% respondents strongly agreed their occupation exposed them to cervical cancer while 28.57% strongly agreed their occupation exposed them to genital warts. Majority of the respondents 32.26%, 41.69%, 32.75% respectively strongly agreed the vaccine should be given to sexually active females, females with multiple sexual partners and all girls before sexual debut. Most of the respondents 65.02% were willing to take the vaccine if they were being offered. Most of the respondents 57.82% strongly agreed to seek the opinion of their health care provider before taking the HPV vaccine. A statistically significant relationship was found between the respondents attitudes and their level of education (p = 0.0198), smoking habit (p = 0.0044) and their duration of sex work (p = 0.0041). Likewise, the proportion of FSW who had good attitude towards HPV infection was significantly higher among those who undertook a Pap smear screening test, compared to those who have not taken the test (p = 0.009) (Table 3).

**Preventive practices towards HPV infection among FSW:** there was an overall good preventive practice (62.28%) among the FSW with a percentage mean practice score of 56.48 ± 27.69 (Table 2). Most of the FSW reported they always have regular check-ups (78.66%) while only 37.22% have once had a Pap smear screening. Among those who reported never having a Pap smear, 33.20% reported as reason fear of abnormal results, 37.15% fear of embarrassment and 29.65% fear of pain. Most FSW opted to take the vaccine for prevention (67.25%). Majority (73.95%) reported the use of condoms when having sex with clients but however just 25.31% reported consistent use of condoms while 48.64% reported occasional use. Among the FSW who reported occasional usage, some had a single reason while others had several reasons. The reasons were unwillingness of clients (30.10%), regular clients (63.78%) and high pay from clients (29.59%). A statistically significant relationship was found between the FSW preventive practices toward HPV infection and their level of education (p < 0.0001), tribe (p = 0.002), marital status (p = 0.0002), former habitat (p = 0.010), their monthly income (p = 0.009) and the religion (p = 0.009).

**Multivariate logistic regression:** after controlling for the confounding effect of the socio-demographic variables, the variables that were significantly associated with the outcomes in the bivariate analysis coupled to other variables were used to run a logistic regression (Table 4). The results showed that participants who were aged 35-51 and 52-68 years were more likely to have good knowledge on HPV than those in the ages 18-34 years (OR = 2.5, p = 0.0001) and (OR = 4.98, p = 0.0565). Respondents who have no educational status are less likely to have good knowledge on HPV compared to those with primary education (OR=0.23, p=0.0077). More so, FSW who lived with their relatives were less likely to have a good knowledge on HPV as compared to their corresponding counterpart who lived alone (OR = 0.33 p = 0.0055). No predictive factor for good attitude was found during the logistic regression analysis. Female sex workers whose based their religion on tradition or other religions were less likely to have a good preventive practice to HPV than their corresponding counterparts who were christians (OR = 0.38 p = 0.0207) and (OR = 0.15 p = 0.0401) respectively. FSW with tertiary education were less likely to have good preventive towards HPV than those having primary education. FSW who were married less likely while those widowed were very more likely to have good preventive practices against HPV (OR = 0.27 p = 0.0142) and (OR = 9.88 p = 0.0013) respectively. However, FSW who lived with friends were more likely to have good preventive practices against HPV infection than their corresponding counterpart who lived alone (OR = 2.17 p = 0.0276).

**Table 4:** multivariate logistic regression analysis

Multivariate logistic regression analysis for the relation between selected factors associated with knowledge on HPV infection among FSWs.			
	OR	95% C I	p-value
**Age group**			
18-34	1		
35-51	2.50	1.58-4.01	0.0001
52-68	4.98	1.11-35.50	0.0565
**Educational status**			
Primary education	1		
Secondary education	1.43	0.87-2.34	0.1592
Tertiary institution	0.75	0.33-1.69	0.4921
No education	0.23	0.07-0.63	0.0077
**Person Living with**			
Alone	1		
Husband	1.89	0.71-5.71	0.2235
Boyfriend	0.61	0.28-1.33	0.2218
Friends	0.84	0.50-1.41	0.5143
Relatives	0.33	0.15-0.71	0.0055
**Multivariate logistic regression analysis for the relation between selected factors associated with Preventive practices on HPV infection among FSWs**.			
	**OR**	**95% C I**	**p-value**
**Religion**			
Christian	1		
Islam	1.01	0.55-1.87	0.9632
Tradition	0.38	0.17-0.86	0.0207
Others	0.15	0.02-0.80	0.0401
**Educational status**			
Primary education	1		
Secondary education	0.99	0.55-1.77	0.9621
Tertiary institution	0.33	0.12-0.85	0.0239
No education	0.78	0.22-2.76	0.6941
**Current Marital Status**			
Single	1		
Married	0.27	0.09-0.74	0.0142
Separated	0.92	0.48-1.79	0.8139
Divorced	1.25	0.57-2.82	0.5892
Widowed	9.88	2.78-48.78	0.0013
**Person Living with**			
Alone	1		
Husband	1.55	0.49-5.08	0.4573
Boyfriend	0.65	0.27-1.49	0.3114
Friends	2.17	1.10-4.39	0.0276
Relatives	0.73	0.31-1.69	0.4613
**Former habitat**			
Rural	1		
Urban	1.61	0.95-2.72	0.0755

## Discussion

There is an overall gap in HPV knowledge among hard to reach populations like FSW. In Nigeria despite the scale up of awareness campaigns in the general population to mitigate the overall risk of HPV infection, FSW population remains an ignored population during these campaigns. Despite several HPV studies conducted in the general population, there is an uncertainty on the level of knowledge on HPV in the FSW community in Nigeria. As to the best of the author’s knowledge no study has been conducted to assess the knowledge, attitudes and preventive practices towards HPV infection in the FSW community. This study was therefore conducted to close this existing gap. Findings of this study revealed a low knowledge and preventive practices with mean score (%) for knowledge 45.68 ± 24.94 and preventive practices 56.48 ± 27.69. This suggests an absolute need to organize awareness campaigns and educative lessons in the FSW community. Findings however demonstrated very good attitudes with a mean score (%) of 72.66 ± 11.61. This is indicative of the fact proper awareness campaigns and educative sessions with this community can markly improve on their knowledge and preventive practice measures. These findings were in accord with studies conducted in Turkey, Thailand and Lima-Peru [20, 22, 23]. Though a slightly higher knowledge was found among FSW in our study compared to those in Turkey, Thailand and Lima-Peru, efforts put in place through health campaigns by non-governmental organizations to decrease the level of STIs in Nigeria could explain this slight difference.

Likewise, the choice of selection site of participants (brothels in our study Vs clinics in the other studies) could impact the choices of answers given as a FSW will feel more at ease to respond while in her brothel than in a general clinic. Contrary to our study, a very low knowledge score of 21.1% was reported in a study in Beijing-China [12]. A knowledge grade of 90% following a post educational intervention on HPV was reported in Penh-Cambodia [24]. This is indicative that setting an appropriate educational intervention in the Nigeria community of FSW could be impacting to their knowledge and preventive practices adopted. Despite the fact most FSW were aware HPV infection was sexually transmissible, less than half were of the view that HPV infection presents itself with vaginal itches/discharge. This could have a poor impact on their preventive practice measures and health seeking behaviour. The use of condom as preventive measures against HPV was equally reported in Peruvian FSW [23]. In our study though most (55.09%) respondents knew an early sexual debut and having more sexual partners could increase their likelihood of having HPV infection, a limited number (14.64%) correctly reported that women who receive HPV vaccine should do a Pap smear. Though just half of the FSW knew early sexual debut and more sexual partners were risk factors for HPV infection, very few were aware of the importance of a Pap smear and vaccination. A similar finding was reported in Beijing-China [12]. This findings re-iterates how important it will be to screen the FSW community for cervical cancer given their high exposure.

Despite the negative and positive attitude portrayed by the general population towards HPV infection, this study found 97.27% of the respondents had good attitudes. This finding is far different from studies found in studies in Thailand who found a negative attitude in the FSW community [22]. The difference in findings is justified by the fact they conducted their study among young FSW while we covered a wide age group. Our results show the FSW knew their occupation predisposes them to several STIs. More so the implication of several NGOs in Nigeria through awareness campaigns on STIs has impacted on the attitudes adopted. This explains their high willingness to seek the advice of a health care worker and consequently their willingness to accept the HPV vaccine if offered. Despite the high positive attitude reported in our study we noticed only a few (37.22%) had actually done a Pap smear with the major reasons being fear of embarrassment, fear of abnormal results and the fear of pain as was reported in a similar study by Kietpeerakool *et al*.in Thailand. Though majority of the respondents (67.25%) in our study were willing to receive HPV vaccination if they were offered, this result is however far lower than the vaccine acceptance level of 97.5% in Lima-Peru [23]. The study conducted in Lima-Peru were informed about cervical cancer which wasn´t the case in our study hence explaining their perceived need to get a vaccine if proposed. FSW in our study reported using condoms either occasionally or consistently and their choice of use depended on high pay from clients, regular clients or unwillingness from clients.

These results are similar to findings from other countries like India and Podgorica-Montenegro [25, 26]. Despite the very high positive attitude seen in our study, it did not reflect the expected practices. The reason could be because attitude is a complex behavioural change pattern that can´t be easily predicted in an individual and it requires several stages of intervention before an individual can adopt a reflective practice. This study found belonging to a higher age group was a significant predictive factor of good knowledge meanwhile being uneducated was a significant predictive factor for poor knowledge. This finding is different from a study conducted in Turkey among FSW where it was found low education and higher age groups were not significant predictors of poor knowledge [20]. Findings from our study implicates being an older FSW increase your chances of being informed on HPV. Though both studies were conducted among brothel based FSW the difference in the findings could arise from the fact that older FSW in Nigeria are more implicated during trainings on STI organised by NGOs. More so the difference could arise from the fact our study sample size almost doubled the study conducted in Turkey. This result is suggestive of the impacts of an external intervention in the level of knowledge. A study conducted on HPV vaccination among female university students in Hong-kong found age was an independent predictor of good knowledge and preventive practices [27]. This can be very implicative in our study as educational based interventions could be very impactful in the FSW community.

Though several factor found to be predictive of preventive practices, to the authors knowledge they did not find in literature studies identifying religion, marital status or living habit as predictive factors of preventive practices. However, finding from this study can be suggestive of the fact FSW who live together turn to discuss on perceive risk they can have following their occupation and this can have an impact in their health seeking behaviour. Our finding is also suggestive widowed FSW will adopt good preventive practices than their married counterpart. This could be highly implicative as married FSW will be at higher risk of STIs hence having a increased risk of transmitting these infections to their partner hence propagating different STIs. The strengths of this study included: to the best of the authors knowledge, it is the first KAP study conducted among FSW on HPV infection. The study was designed following the improvement of the existing questionnaire used to reflect the real tendencies among FSW in the selected study sites. Based on the results found several implicative suggestions have been proposed such that they can align more with the reality found in the field among FSW. Limitations to the study were: closed ended questionnaires were used and this did not allow the FSW to express all their thoughts. Conducting a qualitative study in future among this study population will be more informative. More so, other STIs were not evaluated during the study as this could provide a better understanding of trends most especially between HPV and HIV. This study therefore brings pertinent answers as regards HPV among the FSW community and this could be very implicative in future studies for cervical cancer screening.

## Conclusion

From this study it could be concluded that a good knowledge on HPV infection is very important to effect a good and reflective attitude and consequently a good preventive practices. Because of the influence the clients tend to have on FSW in Nigeria based on our study, the most proper measures put in place to effect and maintained good practice will be very necessary not only in the female sex worker community but equally in the general population. With several factors being predictive on the level of knowledge or preventive practice, there is therefore a need for knowledge improvement through health education interventions among the hard to reach populations like the FSW. Because FSW constitute a very heterogenous population and taking into account their risk behaviours vary across age groups and occupational venue, all prevention interventions programs ought to be tailored to the particular sub population. Therefore a high need for the Federal Ministry of Health in Nigeria to organise and promote health education awareness campaigns targeted on the FSW community on STIs with much emphasis on HPV infection and to promote vaccine uptake and Pap smear screening by subsidizing the cost. NGOs should continually be more engaged in health awareness campaigns among the FSW but laying more emphasis on HPV alongside HIV.

### What is known about this topic

- Studies of knowledge on HPV has been conducted among secondary school girls and health care workers in tertiary hospitals in Nigeria.

### What this study adds

- As to the best of the authors knowledge, this study is the first conducted among female sex workers in Nigeria;
- It scored and graded attitudes and preventive practices which other studies did not;
- It went further to evaluate the factors that could be responsible for the observed knowledge, attitudes and preventive practices.
